# Hydrogen Storage, Magnetism and Electrochromism of Silver Doped FAU Zeolite: First-Principles Calculations and Molecular Simulations

**DOI:** 10.3390/polym11020279

**Published:** 2019-02-07

**Authors:** Wei-Feng Sun, Yu-Xuan Sun, Shu-Ting Zhang, Ming-He Chi

**Affiliations:** 1 Heilongjiang Provincial Key Laboratory of Dielectric Engineering, School of Electrical and Electronic Engineering, Harbin University of Science and Technology, Harbin 150080, China; yuxuan_hrbust@126.com (Y.-X.S.); shuting_hrbust@126.com (S.-T.Z.); minghe_chi@126.com (M.-H.C.); 2 Key Laboratory of Engineering Dielectrics and Its Application, Ministry of Education, Harbin University of Science and Technology, Harbin 150080, China

**Keywords:** hydrogen storage, silver cluster, FAU zeolite, first-principles calculation, molecular simulation

## Abstract

The complex configuration, H_2_ adsorption binding energy, magnetic, and optical properties of FAU zeolites with Ag cations loaded by ion exchange in the vacant dielectric cavities were investigated by employing the first-principles calculations with all-electron-relativistic numerical atom-orbitals scheme and the Metropolis Monte Carlo molecular simulations. The visible absorption spectrum peaked at distinct wavelengths arranging from red or green to blue colors when changing the net charge load, due to the produced various redox states of Ag cations exchanging at multiple Li^+^-substituted sites. The spin population analyses indicate the ferrimagnetic coupling between Al–O–Si framework and Ag cations originates from the major ferromagnetic spin polarization in Ag cation cluster coordinating with sodalite cages, with the net spins appreciably depending on the Ag content and exchange site. The H_2_ adsorption capacities and binding energies represent significant dependence on the content, location, and electronic property of Ag cations introduced into the FAU zeolites. The evident decrease of H_2_ adsorption binding energy with increased loading concentration demonstrates repulsive interaction between H_2_ molecules and heterogeneous adsorption configuration on Ag cations. The adsorption sites of H_2_ sorted by the binding energy with different adsorption configurations were correlated with exchange sites of Ag cations under different Ag loading to comprehend the H_2_ adsorption mechanism.

## 1. Introduction

The transition metal (TM) cations introduced by exchanging Si or Al covalent atoms with lower valence state atoms of III or II main group in zeolite framework at specific porous locations can present specific physical-chemistry environment due to the characteristic metal-framework interaction and dominate the adsorption and heterogeneous catalysis performance [1]. Recent studies primarily focus into controlling reactivity and exploring the correlated mechanism especially for the octahedral zeolites (faujasites) [1,2,3]. The octahedral framework structure of faujasites with nanoscale pore is constructed by eight sodalite cages that are connected by oxygen bridges between hexagonal faces, so as to build a large central supercage with a cavity of ~12 Å diameter and 12 faces of atomic ring with 7~8 Å diameter [4,5]. It has been demonstrated that the decorated TM cations prefer to locate inside the supercages with a diameter of ~13 Å in hydrated faujasites which can provide adequate space to accommodate hydrated cation sphere capped with different quantities of water molecules determined by cation attribute [6]. Although the silver-exchanged zeolites have been extensively studied in terms of the catalytic properties, the small Ag^+^-cluster zeolites are still of great interest in zeolite cavity, nanoparticles and clusters synthesized by size controllable technique [7]. Especially, Ag-exchange linde type-A (LTA) or faujasite (FAU) zeolites are capable of emitting different fluorescent after celcination, resulting from the formation of multiple clusters such as Ag_3_*^n^*^+^ and Ag_6_*^n^*^+^ etc. in zeolite cages by autoreduction [8,9]. In the Ag^+^ reduction process that the silver ions loaded in porous zeolites are being reduced to form charged silver clusters by dehydration, the electrons transfer primarily from the oxygen atoms in the framework or hydration water which will be oxidized to oxygen molecules. Furthermore, it has been demonstrated that the color of Ag doped zeolites can change from white through yellow to rick red or black due to multiple ionic silver complexes during the dehydration, while with sterile fluorescent characteristics at ambient temperature [8]. Up to now, the essential mechanism of silver cations formation in the porous zeolite structures has not been well comprehended and precisely pictured, which also play pixel role in understanding and improvement of gas storage and electrochromism especially when the silver cluster migrate between different locations at variable environment such as temperature and applied electric field. To our knowledge, although it has been suggested from experimental investigations that considerable magnetism and electrochromism could be introduced into lithium-exchanged low-silica X-type (Li-LSX) zeolites by doping TM [9], no *ab initio* theoretical study has been reported for TM-exchange Li-LSX zeolites to calculate the spin-polarized electronic structure, based on which the magnetic and optical properties will be predicted and the underlying physics can be revealed.

The high interest of exploring solid materials for hydrogen (H_2_) storage lies in the ideal candidate energy resource of great earth-abundant H_2_ without carbon pollutants to replace fossil fuels, since their oxidation engenders only the by-product of water. More important, it is primary to capture H_2_ and storage them in solid media which is being as a fuel carrier to fulfill the H_2_/O_2_ solid electrolyte fuel cell. The highly efficient chemical energy in comparison to minimal molecular mass of hydrogen (143 MJ/kg) is more than three times as much as that of gasoline (44.4 MJ/kg), while hydrogen energy content to gas volume is much lower (0.0108 MJ/L) compared with liquid gasoline (34.8 MJ/L) in ambient condition [10]. Therefore, hydrogen storage by capturing gas H_2_ with high uptake is the most challenge to apply hydrogen as an alternative fuel. Previous studies have implied that hydrogen storage by assembling H_2_ into liquid phase loaded in tanks under high pressure is not yet efficient due to the low ratio of energy content to volume and the high producing cost. With a novel method initialized by adsorbing interaction between hydrogen and porous materials that serves as storage media, it is critically important to develop new nano-structured materials that is pertinent to absorbing hydrogen with high gravimetric and volumetric densities. The hydrogen storage materials should be consisting of light elements and with large surface area. It is recently reported that the nanoporous materials decorated with metallic atoms (transition or alkali metals) represent excellent uptake capability of molecular H_2_ [11]. The metal atoms decorating in nanoporous structures play a key role in molecule adsorption, which result in significantly enhanced storage capacity of H_2_ with desirable adsorption energies. In terms of theoretical/computational researches, molecular simulations with Monte Carlo method have been performed in order to predict the preferable sites, isotherms and isosteric heat of adsorption, as well as to investigate the separating mechanism for atmosphere gas binary mixtures in Li-LSX zeolites [12]. The recent literature reviews indicate little Monte Carlo research, especially for the equilibrium adsorption properties of N_2_, O_2_ and H_2_ in nanoporous Li-LSX zeolites [4,13].

The present study try to theoretically explore the magnetic resource, the visible spectrum change of optical property under charge injection (electrochromism), the storage capacity of H_2_ in essential mechanism of Ag cluster formation and distribution for Ag-exchange Li-LSX zeolites. The Ag concentration, FAU framework topology and charge population are correlated to analyze the gas adsorption mechanism by first-principles calculated adsorption bind energy together with adsorption isotherm from molecular simulation with Monte Carlo method. The electrochromism induced from multiple Ag-cluster complex is investigated by energy-minimized geometry optimization for Ag cation aggregation and optical property from first-principles electronic structure calculations.

## 2. Theoretical Methodology

The atomic structure models of Li-LSX FAU zeolites are constructed based on the reported theoretical and experimental data of lattice constant and atom coordinates [4,5]. The Li^+^ cations at various sites with different symmetrical attributes in Li-LSX unit cell are individually substituted by Ag cations to model Ag-exchange Li-LSX (Ag*_x_*Li_1−*x*_-LSX) zeolites, which will be as the initial structure for further geometry optimization of first-principles total energy calculations. The atomic and charged Ag-cluster configurations, magnetic property, optical properties and H_2_ molecule adsorption binding energy of Ag*_x_*Li_1−*x*_-LSX FAU zeolites are calculated by all-electron and numerical atom-orbitals first-principles electronic structure scheme as implemented in DMol3 program of Materials studio 8.0 software package [14,15], to investigate the mechanisms of Ag cluster formation, magnetic polarization, electrochromism and to analyze the H_2_ adsorption interaction and uptake performance. Metropolis Monte Carlo (MMC) molecular simulations with Sorption program are implemented to calculate adsorption isotherm for H_2_ in Ag-exchange Li-LSX zeolites with FAU framework.

The first-principles electronic structure calculations are performed utilizing all-electron numerical orbital basis set method by DMol3 program with GGA exchange-correlation functional in PBEsol form [16]. The electron wave-functions of intrinsic states are expended by double numerical polarized (DNP) basis set with global orbital cutoff being set as 5.0 Å to adequately reduce the error induced from finite basis set. The introduced Ag atom with 4*d* electrons may cause spin-splitting or single electron occupied states with local and net spin, the spin-polarized calculations are therefore performed using different orbitals for different spins based on the spin density functional theory applying Dirac relativistic quantum mechanical equations to include spin-polarization interaction [17]. The interaction between electrons and atomic core are described by all electron relativistic core treatment. The convergence toleration for self-consistent field (SCF) iteration is set as 1.0 × 10^−6^ Ha/atom (1Ha = 27.2 eV), and DIIS density mixing scheme with charge and spin mixing amplitudes of 0.2 and 0.5 respectively is used to relax electrons [18]. The 0.002 Ha thermal smearing of orbital occupation is employed to expedite SCF convergence. The ***k*** point sampling of Brillouin zone integration is carried on Monkhorst-Pack 4 × 4 × 4 grid. The geometry optimization of total energy functional minimization is implemented with conjugate gradient algorithm to obtain convergence quality of energy, force and displacement lower than 1.0 × 10^−5^ Ha, 0.002 Ha/Å and 0.005 Å respectively [19]. The dispersion correction of exchange-correlation functional is used to include weak dispersion interaction [20]. The nonpolarized optical properties are calculated by the time-dependent density functional theory (TD-DFT) excitation method using adiabatic local density approximation (ALDA) kernel with exchange-correlation terms included [21]. In the MMC molecular simulations as implemented by Sorption program to represent gas adsorptions, the COMPASS II forcefield is adopted with the electrostatic potential and van der Waals interactions being calculated through Ewald & Group and Atom based summations respectively, and the equilibration and production steps are set as 1 × 10^6^ and 1 × 10^7^ respectively [22,23].

## 3. Results and Discussion

### 3.1. Atomic Structure

**Figure 1:**
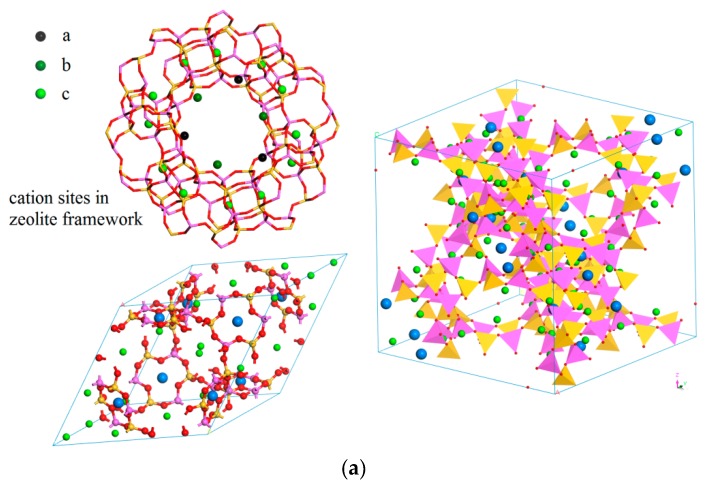

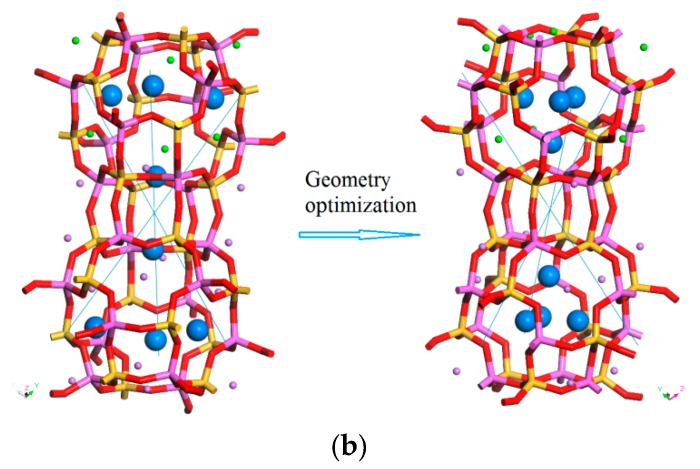
(**a**) The geometrically optimized structure of aAg_0.29_Li_0.71_-LSX in primitive (**left**) and traditional (**right**) unit cells with FD-3 space symmetry, with the three types of Li cation sites being identified by a, b, and c shown in the zeolite framework above primitive cell; (**b**) Notable transfer of Ag cation to the optimized location into sodalite cage by geometrical optimization for cAg_0.29_Li_0.71_-LSX. The blue, green, pink, yellow, and red balls represent Ag, Li, Al, Si, and O atoms respectively. The pink and yellow tetrahedrons symbolize Al–O and Si–O framework configurations respectively shown in right panel.

According to the space symmetry of Li-LSX in FAU zeolite crystallinity, the Li^+^ cations can be discriminated into three types based on their locations with different chemical environments as indicated by a, b, and c in Figure 1a, which are respectively and gradually substituted by Ag cations so as to build-up six representative species of Ag-exchange Li-LSX (Ag*_x_*Li_1−*x*_-LSX) structures with nomenclature by the kinds of replaced Li cations as listed in Table 1. The a and b sites inside supercage are ascribed to the central positions in front of framework 4-ring and 12-ring respectively, while site c is located at the center of triangular joint interface between sodalite cage and hexagonal prism. The Ag-exchange Li-LSX is formed in crystallographic structure of FD-3 space symmetry with the lattice constant increasing as the increment of Ag content and a fixed lattice angle of 60°, as the parameters shown in Table 1 in which the lattice constant and the bond length of Ag-O coordination are listed for the geometrically optimized structures. The zeolite framework structure of Ag-exchange Li-LSX is made up of eight sodalite cages, between and in which the Ag cation and Ag-cluster are interacting with them by ionic bond and coordination bond respectively, so as to construct a nanoporous structure with a supercage of ~14 Å diameter, as shown in right Figure 1. The geometrical optimization from first-principles energy minimization indicates that the cell dimension not only increases slightly with Ag doping content due to large ionic radius of Ag cation, but also depends on the Ag occupying sites (a, b or c) of ion exchange for substituting Li cation. Especially, after geometrical optimization, the Ag cations exchanged on c site initially being at the center of joint interface between between sodalite cage and hexagonal prism in cAg_0.29_Li_0.71_-LSX have transferred into sodalite cage, as to form a tetrahedron cluster of four Ag cations (Ag_4_*^n^*^+^) with substantial coordination to sodalite cage, as shown in Figure 1b. Further for aAg_0.29_Li_0.71_-LSX, the Ag cations at the a-type sites protrude out from the sodalite cage surface inwards supercage pore, being in favor of binding multiple molecules around Ag cation. Due to the larger radius of Ag cation than that of Li cation, the distance *d*_Ag-O_ between Ag cations and covalent O atom is 26% and 22% longer in aAg_0.29_Li_0.71_-LSX and bAg_0.43_Li_0.57_-LSX respectively than the distance *d*_Li-O_ between Li^+^ and O atom in Li-LSX, while the considerably lower total energy with a reduced electrostatic component achieved by exchanging Ag on c site as for cAg_0.29_Li_0.71_-LSX with a smaller lattice constant implies the significant coordinating interaction of Ag_4_*^n^*^+^ to Al-Si-O framework by providing high density of multiple Ag 4*d*-orbitals. The exchange Ag to the a or b sites inside supercage, which interact out of the sodalite cage and hexagonal prism through a component of Coulomb force, have little effect on Al-O-Si structure for aAg_0.29_Li_0.71_-LSX, bAg_0.43_Li_0.57_-LSX, in comparison to the Ag_4_*^n^*^+^ coordinating in sodalite cages for cAg_0.29_Li_0.71_-LSX. The calculated lattice parameters of Li-LSX are in less than 3.0% difference with the experimental results, demonstrating the reasonable scheme and accuracy of results in our calculations.

### 3.2. Electronic Structure

**Figure 2:**
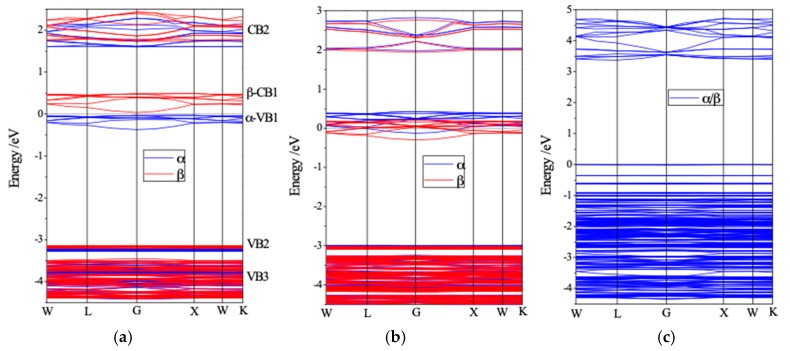
Calculated band structures of (**a**) aAg_0.29_Li_0.71_-LSX, (**b**) bAg_0.43_Li_0.57_-LSX, and (**c**) cAg_0.29_Li_0.71_-LSX. The energy is referenced by setting Fermi energy level as zero. VB: valence band; CB: conduction band.

The electronic structures of Ag-exchange Li-LSX structures obtained by geometric optimization of energy minimization are calculated with a highly symmetric energy dispersion path of W-L-G-X-W-K in Brillouin Zone is adopted. The calculated electronic energy dispersion relation *E*(***k***)-band structure, density of electronic states (DOS) and atomic projected density of state (PDOS) of three fundamental doped structures which are adequate to render the essential electronic properties of all the six type of Ag-exchange Li-LSX are illustrated in Figure 2. The Ag-exchange Li-LSX represents the semiconductor character of band structure, with band-gaps of photoelectronic transitions as listed Table 2. The electron spin polarization leads to the obvious spin-splitting in the energy band near the Fermi energy level. The up (*α*) and down (*β*) spin states shifts towards the low and high energy respectively, resulting in evidently higher number of up-spin electrons in valence band which engenders magnetic moment of net spin in Ag exchange Li-LSX.

It is noted from Figure 2 that the valence band of aAg_0.29_Li_0.71_-LSX is mainly composed of three non-overlapping bands: VB1, VB2, and VB3. The lowest 1.15 eV width of energy band (VB3) is 0.22 eV below the VB2 which are almost horizontal without dispersion and to a large distance (2.77 eV) below the highest valence band of parabolic *α*-VB1. The band structures of Ag*_x_*Li_1−*x*_-LSX render discrepant characteristics conduction band for different Ag content and exchange sites, and show appreciable spin-splitting from spin polarization near Fermi level with higher levels of spin-down states than spin-up states, consequently splitting into two spin bands of *α*-VB1 and *β*-CB1. Compared with aAg_0.29_Li_0.71_-LSX, bAg_0.43_Li_0.57_-LSX represents a similar character of band structure but with inverse spin-splitting, due to the almost identical interactions of Ag cations on a and b sites coordinating with framework. In contrast, no energetic spin-splitting of electronic eigenstates for cAg_0.6_Li_0.4_-LSX is observed, illustrating the downwards parabolic CB with minimum at L point and the horizontal VB without dispersion as shown in Figure 2c. The Ag cation cluster at c site contribute no spin-polarization, due to the intensive coordinating from the electrons provided by Al-O-Si framework to the Ag-4*d* orbitals which results in spin-pair states without spin-polarization. The discrete valence bands near Fermi level come from the coordination bonds of Ag cation cluster with the sodalite cage. In the band structure of aAg_0.29_Li_0.71_-LSX and bAg_0.43_Li_0.57_-LSX, notable spin splitting occurs in the CB2 with an obvious upward parabolic dispersion at G point. Both the valence band maximum (VBM) and conduction band minimum (CBM) locate at G point, illustrating that Ag exchange Li-LSX are direct band-gap semiconductors. The photoelectronic band-gap values of Ag-exchange Li-LSX except for c site Ag-exchange materials are in the range of 1.70~2.16eV, as listed in Table 2, which can fabricated by proper doping technique to the n or p-type semiconductor materials that are electrically sensitive to visible light.

**Figure 3:**
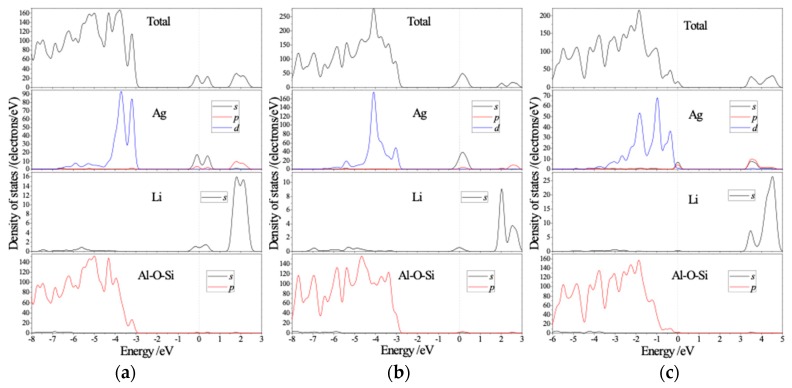
Calculated total and atomic projected density of states (DOS) for (**a**) aAg_0.29_Li_0.71_-LSX, (**b**) bAg_0.43_Li_0.57_-LSX and (**c**) cAg_0.29_Li_0.71_-LSX. The panels from up to down represents total DOS and partial projected density of state (PDOS) of Ag, Li and Al-O-Si framework atoms respectively. The Fermi energy level is set as the reference energy zero (vertical dash line).

From the the total density of states (DOS) and the atomic projected density of states (PDOS) as shown in Figure 3, the orbital-component contributing from each atom to energy band can be observed: the lower valence band VB2 is composed of Ag 4*d*-orbitals (Ag-4*d*) and 2*p*-states from Al-O-Si framework; VB1 is mainly derived from the *s*-orbitals of Ag and Li atoms, and a minority of Ag 4*p*-orbitals (Ag-4*p*); VB3 consists of the 2*p*-state electrons of Al, Si, and O atoms. The higher conduction band (CB2) of Ag-exchange Li-LSX is dominantly contributed by Ag 4*p*-orbital and Li 2*s*-orbital. When the content of substituted Ag ions in Ag-exchange Li-LSX increases, the low energy valence band originating from Ag-4*d* increase appreciably in DOS, indicating the hybridized electronic states of Ag-4*d* coordinating with 2*p*-states of Al-O-Si framework. The CBM characteristics of all these zeolites are dependent on Ag-4*p* states. With the increase of Ag content, these Ag *p*-states shift to higher energy, resulting in larger photoelectronic band-gap.

### 3.3. Magnetic Property

**Figure 4:**
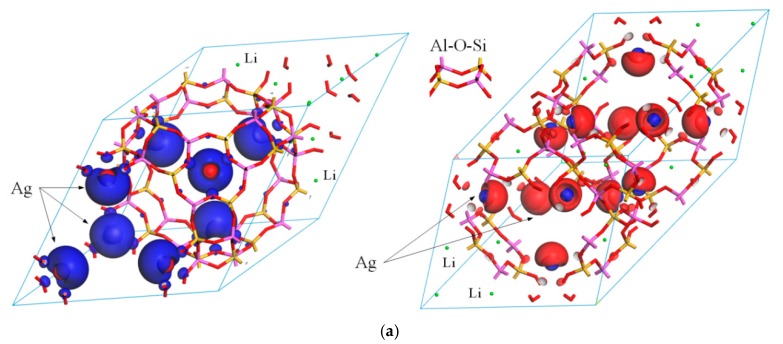

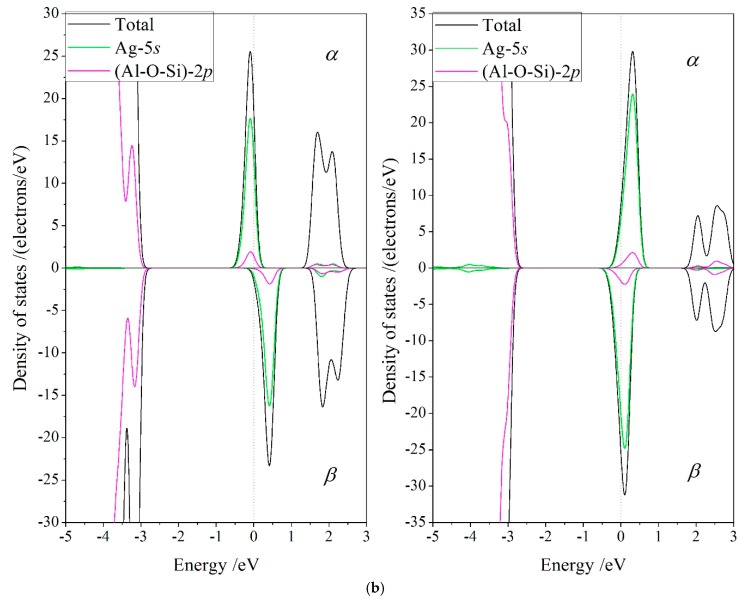
(**a**) The spin distribution representing as spin density isosurface contoured at 0.005 electrons/Å^3^ with blue and red colors represent dominant spin states of *α* and *β* respectively; (**b**) the spin-resolved partial PDOS of Ag-5*s* and framework 2*p*-states with Fermi energy level being referenced as energy zero indicated by vertical dashed line, for aAg_0.29_Li_0.71_-LSX (**left**) and cAg_0.29_Li_0.71_-LSX (**right**).

Due to similar framework circumstance near the exchange Ag cations, the aAg_0.29_Li_0.71_-LSX and bAg_0.43_Li_0.57_-LSX will represent similar magnetic property resulting from sin polarization, which has been verified from calculated electronic structures in previous section. Hence, only the calculated results of aAg_0.29_Li_0.71_-LSX and bAg_0.43_Li_0.57_-LSX are emblematically presented here, as shown in the Figure 4 illustrating the space distribution of spin density isosurface and the spin-resolved partial DOS. Since the net magnetic moment dominantly origins from the doped Ag, the magnetic performances of the other four piece of Ag-exchanged Li LSX will correspondingly be the additive results of aAg_0.29_Li_0.71_-LSX and bAg_0.43_Li_0.57_-LSX with individual Ag substitution weight.

The band structure and DOS have demonstrated that the electron eigenstates of Ag-exchange Li-LSX have obvious spin-splitting near Fermi energy level except for cAg_0.29_Li_0.71_-LSX, implying that the spin symmetry of *α* and *β* electronic states has been destroyed. The spatial distribution of spin density isosurface in aAg_0.29_Li_0.71_-LSX as shown in left panel of Figure 4a indicates that spin polarization results in asymmetric distribution of spin states in space. The major *α* and minor *β* electrons are distributed around the outward and interval spaces of Ag cation from the Al-O-Si framework respectively. The remarkably higher density of *α* states than that of *β* states implies substantial ferromagnetic spin polarization between Ag cations and leads to the considerable net spin (magnetic moment) with ferrimagnetic spin coupling between zeolite framework and Ag cations. In comparison, bAg_0.43_Li_0.57_-LSX represent inverse spin polarization characteristics indicating that *α* and *β* electrons lose their spatial symmetry (as shown in the right panel of Figure 4a) with the major *β* electrons being distributed on the ferromagnetic coupled Ag cations in closer coordination with Al-O-Si framework, resulting in relatively lower net spin, as listed in Table 2. It is thus suggested that the magnetism of Ag-exchange Li-LSX originates from Ag ion exchange, based on which the magnetic moment could be under artificial control.

Besides the spin-polarization caused asymmetric distribution of different spin states in space, the density of spin-up (or spin-down) electron is obviously higher than that of spin-down (or spin-up) electron due to the splitting of spin energy levels, which is identical to the spin polarization results of ferromagnetic coupling between Ag cations as mentioned above. The produced net spin is majorly distributed around the Ag cations due to the ferromagnetic spin coupling of adjacent Ag ions, which is intrinsic source of magnetism in Ag-exchange Li-LSX. The results of partial PDOS (distinguishing up and down spin states) of Ag-exchange Li-LSX are shown in Figure 4b. It is further confirmed that the spin splitting of electron intrinsic states near Fermi energy level is essentially due to the spin polarization of Ag 5*s*-orbitals (Ag-5*s*).

### 3.4. Optical Properties

The optical properties generated by electron excitation are calculated by DMol3 program, in which the the complex refractive index *N* = *n* + *ik* and absorption coefficient *η* are derived from the calculations of complex dielectric constant *ε* = *ε*_1_ + i*ε*_2_. The optical absorption spectra can be obtained by the relations *ε*_1_ = *n*^2^ − *k*^2^*ε*_2_ = 2*nk* and *η* = 2*kω* (*ω* denotes the angular frequency of incident light). In calculating the optical properties, the propagation and polarization directions of incident light can be defined. Because Ag-exchange Li-LSX has high point symmetry in three orientations and the color characteristics of the material is determined by the optical property of non-polarized light, the non-polarized light is defined as being incident along the [001] crystallographic direction of Ag exchange Li-LSX, meaning that the electric field vector of incident photon distributes uniformly in all directions of (001) crystallographic plane. The formula for calculating the imaginary part of complex permittivity by DMol3 is as follows:

(1) $$\varepsilon 2(q\to Ou,h\omega )=\frac{2\pi e^{2}}{\Omega \varepsilon 0}{\sum_{k,v,c}\left|\langle \psi_{k}^{c}\left|u\cdot r\right|\psi_{k}^{v}\rangle \right|}^{2}\delta (E_{k}^{c}-E_{k}^{v}-E)$$

where **u** symbolizes the vector defining the polarization direction of the incident electric field, the superscript c and v identify the conduction band and the valence band respectively. This expression is similar to the Fermi’s Golden rule for time-dependent perturbations. The *ε*_1_(*ω*) thus can be deduced by the Kramers–Kronig transformation between real and imaginary parts of complex permittivity. The matrix elements of position operator needed to describe the electron transition in the above equations can be written as the matrix elements of momentum operator which can be calculated directly in the reciprocal space as by:

(2) $$\langle \psi_{k}^{c}\left|u\cdot r\right|\psi_{k}^{v}\rangle =\frac{1}{i\omega m}\langle \psi_{k}^{c}\left|P\right|\psi_{k}^{v}\rangle +\frac{1}{\hbar \omega }\langle \psi_{k}^{c}(\left|V_{nl}\right|,r)\psi_{k}^{v}\rangle$$

where **P** represents momentum operator, and m is electron mass.

**Figure 5:**
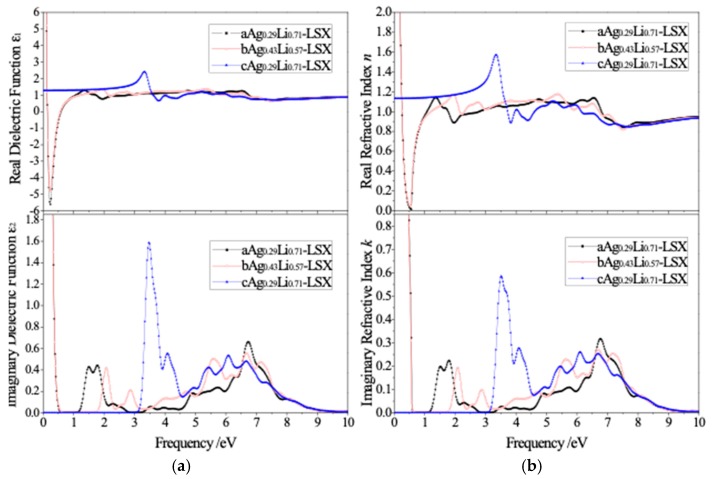
The calculated spectra of (**a**) dielectric function and (**b**) optical refractive index for Ag-exchange Li-LSX with the unpolarized light being incident along [001] crystallographic direction.

The complex dielectric function and refractive index of Ag-exchange Li-LSX are calculated according to calculated results of electronic structure, as the plotted spectra of real part *ε*_1_/*n* and imaginary part *ε*_2_/*k* shown in Figure 5. The calculated values of the static dielectric constant *ε*_0_ and refractive index *n*_0_ increase with the increase of Ag doping content. There are several spectral peaks in the 0–10 eV frequency range for the dielectric function and refractive index spectra, and the fluctuation amplitude of spectrum line decreases with the increasing frequency, approaching to stable values after exceeding 10eV. aAg_0.29_Li_0.71_-LSX and bAg_0.43_Li_0.57_-LSX are very close in the spectra of dielectric function and refractive index, while one special peak with relatively higher intensity appears in the frequency range of 3.0–3.5 eV for cAg_0.29_Li_0.71_-LSX in contrast, indicating the optical properties are dominantly determined by Ag exchange site, not by the Ag content. The spectral peaks of imaginary dielectric function and refractive index correspond to the electron excitation from valence band to conduction band, according to which the electron transition can be analyzed.

**Figure 6:**
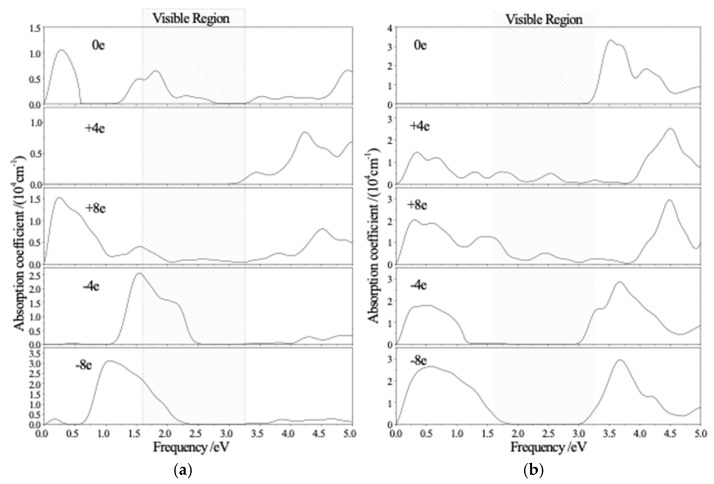
The calculated optical absorption spectra of (**a**) aAg_0.29_Li_0.71_-LSX and (**b**) cAg_0.29_Li_0.71_-LSX for the unpolarized light incident along [001] crystallographic direction, under different charge loading (−8e~+8e per unit cell).

The peaks of imaginary dielectric function is mainly derived from the electron transition from *p*-state electrons of Al-O-Si framework in valence band to the Ag-4*p* and Li-2*s* states in conduction band. In order to study the electrochromic performance, the optical absorption spectra of Ag-exchange Li-LSX charged with −8e~+8e/unit cell (averaged 0.25e~1.0e Ag cations, e is elementary charge) are calculated as the results shown in Figure 6. Under electric neutrality without charge loading, there appear characteristic absorption peaks in red visible (1.8 eV) and far infrared (0.27 eV) region for aAg_0.29_Li_0.71_-LSX, while only two explicit peaks with higher and lower intensities of 3.5 and 1.8 × 10^4^ cm^−1^ locate at ~3.6 and ~4.2 eV in ultraviolet region for cAg_0.29_Li_0.71_-LSX with the cutoff frequency of 3.2 eV in visible region. Especially, the spectral characteristics (cutoff frequency, peak position, and intensity) in visible region alternate appreciably with variation of charge loading (shadow region in Figure 6) and engender absorption peaks appearing in infrared band. Furthermore, the intrinsic absorption band in the lowest frequency approaching to zero for aAg_0.29_Li_0.71_-LSX originates from the electronic transition between different spin states near Fermi level caused by magnetic oscillation of incident electromagnetic field. After loading charges in aAg_0.29_Li_0.71_-LSX, the two absorption peaks in lower frequency than purple limit disappear and also recur obviously with increasing positive charge load, while the far-infrared absorption decline and the distinctive peak in the region from near-infrared to red visible region apparently increase in intensity and spread abroad when negative charges are introduced, reducing the optical cutoff frequency from 1.2 eV to <0.5 eV. When cAg_0.29_Li_0.71_-LSX being positively charged, multiple discrete absorption peaks appear in visible and infrared regions with the intensity increasing with the increment of charge quantity. Nevertheless, only a broad infrared peak is presented when loading negative charges in cAg_0.29_Li_0.71_-LSX, and the absorption cutoff frequencies of this novel absorption band and the intrinsic ultraviolet peak extend into visible region with the increase of charge loading. In particular, discriminated absorption peaks in red and green-blue (2.4−2.7 eV) visible regions will be represented under positively and negatively charged conditions respectively for Ag-exchange Li-LSX with a and c sites of Ag cations.

When the Ag-exchange Li-LSX is positively charged, the valence band-edge electrons of Ag-5*s* and Ag-4*p* states are ionized (forming holes) and thus leads to the transition of Ag valence states to higher oxidation states (the different redox states of Ag cations show different colors). Therefore, the optical absorption spectrum changes obviously in the visible region with varying charged states. When the system is negatively charged, the virtual electronic states of conduction band bottom, which also dominantly derive from the contribution of Ag 4*p* and 5*s* orbitals, is occupied by electrons so as to transfer the Ag valence states to lower oxidation states. The doped Ag ion will alternate between its multi-redox states rendering different visible colors with the variation of charging states, resulting in a corresponding change in optical properties of Ag-exchange Li-LSX. Under the analogic mechanism, the hole and electron carriers generated in valence and conduction bands respectively by p-type and n-type doping will also make the optical properties of Ag-exchange Li-LSX change similarly and show discriminated colors with different charged states. Color control can be fulfilled by semiconductor doping technique. Further, the light absorption in the infrared region under charged condition originates from the in-band (intraband) transition of hole or electron. The calculated results of the above optical properties demonstrate that the Ag-exchange Li-LSX have distinctly characteristic colors in visible region, and the absorption spectra represent remarkable electrochromism. Besides, Ag-exchange Li-LSX has high absorptivity in ultraviolet region and thus can also be exploited for UV photoelectron detectors.

**Figure 7:**
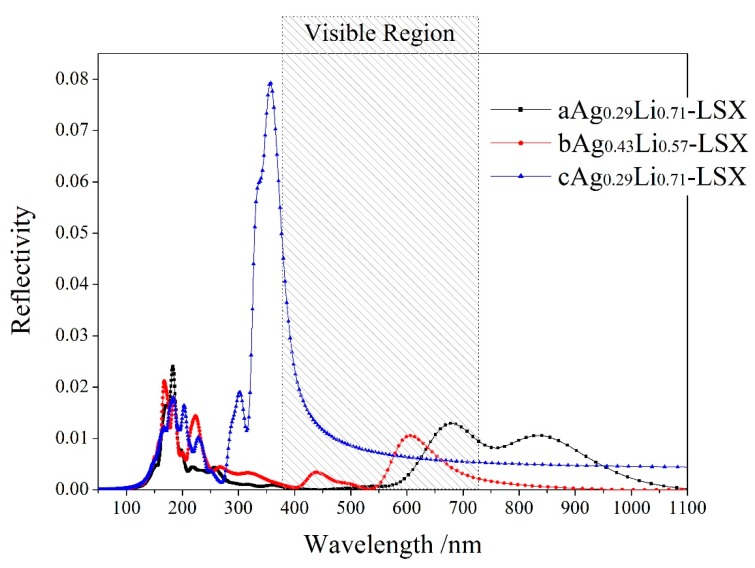
The calculated optical reflectivity spectra of Ag-exchange Li-LSX with the unpolarized light being incident along [001] crystallographic direction.

The calculated spectral lines of optical reflectivity varying with incident wavelength for Ag-exchange Li-LSX are shown in Figure 7, indicating no reflection occurs in the wavelength range of <50 nm and almost zero reflectivity in visible region (<0.04). aAg_0.29_Li_0.71_-LSX and bAg_0.43_Li_0.57_-LSX are very similar in optical reflection: two minimal reflection bands with tiny peak values of 0.01–0.02 distribute in 150–250 nm and 550–900 nm. The reflectivity spectrum of cAg_0.29_Li_0.71_-LSX shows similar character in the <270 nm range and an extraordinary peak of 0.08 at 350 nm which rapidly decreases in red visible region and declines monotonously to a constant value of ~0.005 in 670–1100 nm. With the increase of Ag content, the spectral lines of reflectivity is red shifted with the peak intensity being notably reduced. Conspicuous optical reflection occurs in ultraviolet region with the little reflectivity varying in the range of 0.005–0.025 and 0.01–0.08 for exchanging Ag on a/b and c sites respectively. The difference of optical properties is due to the different electronic structures resulting from the coordinating interactions of Ag cations in different content and exchange sites. These results provide reliable theoretical basis and data support for the development of new optoelectronic materials and devices.

### 3.5. H_2_ Storage Capability

**Figure 8:**
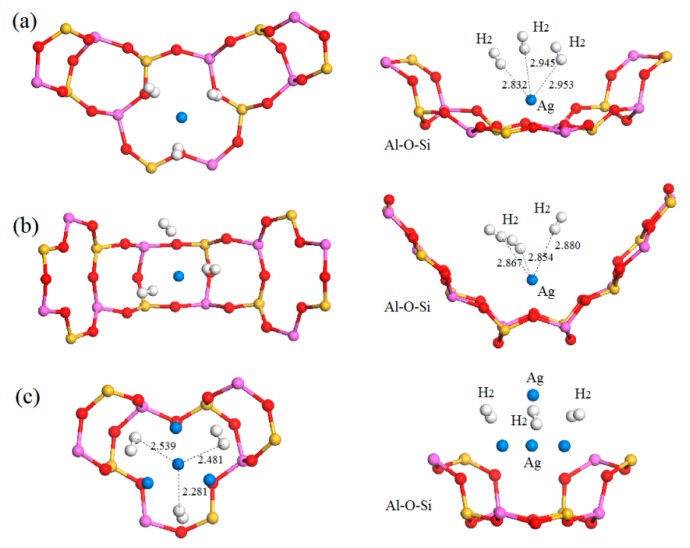
Optimized adsorption configurations and corresponding binding energies of H_2_ molecule in (**a**) aAg_0.29_Li_0.71_-LSX, (**b**) bAg_0.43_Li_0.57_-LSX, and (**c**) cAg_0.29_Li_0.71_-LSX. The indicated distance from the adsorbed H_2_ molecule to the doped Ag cation is measured in Å. The green, pink, yellow, red, and white balls represent Li, Al, Si, O, and H atoms respectively.

Through the interaction of transition metal Ag cation with the frame surface, H_2_ molecules can be substantially absorbed into the Ag-exchange Li-LSX with higher binding energy and more adsorption sites, as shown in Figure 8 for the geometrically optimized configurations of three H_2_ molecules adsorption in aAg_0.29_Li_0.71_-LSX and cAg_0.29_Li_0.71_-LSX, where the absorbed H_2_ molecules are binding to the Ag cations in and between zeolite cages respectively. It is easy to lose *d*-orbital electrons for the Ag cations that substituting Li cations on a or b site, and the electrostatic potential energy of the interaction with H_2_ will be higher, compared with Ag cations on c site where the absorbed H_2_ molecules while bear more absorbing attraction from the sodalite cages and hexagonal prism of Al-Si-O framework. The binding energies of a single H_2_ adsorbed around the Ag cations on the exchange sites of a, b and c in Ag-exchange Li-LSX are 0.492, 0.449, and 0.634 eV respectively with the distances of 2.1~2.4 Å to Ag cation, which are slightly lower and explicitly higher than the lower limit of chemisorption energy (~0.52 eV). The chemisorption of H_2_ molecules around Ag cations indicates that the H_2_ capture performance of Li-LSX can be improved significantly by Ag doping, but it will be difficult to be desorbed as the physical adsorption. Ag cation exchange will apparently increase the H_2_ storage capability of Li-LSX. However, the binding energy between H_2_ and Ag cations at c sites exceeds the lower limit of chemisorption energy, which makes it difficult for H_2_ molecules to be desorbed. Meanwhile, the interactions between H_2_ and Ag cations at a and b sites are between physical and chemical adsorptions, and thus the reversible process of H_2_ adsorption and desorption at ambient temperature can be realized by appropriately regulating Ag cation substitution. In order to practically apply the ameliorated H_2_ storage capability of Ag-exchange Li-LSX, specific chemical or molecular techniques for doping process in material preparation should be employed to intensively replace Li cations at a or b site with Ag cations.

**Figure 9:**
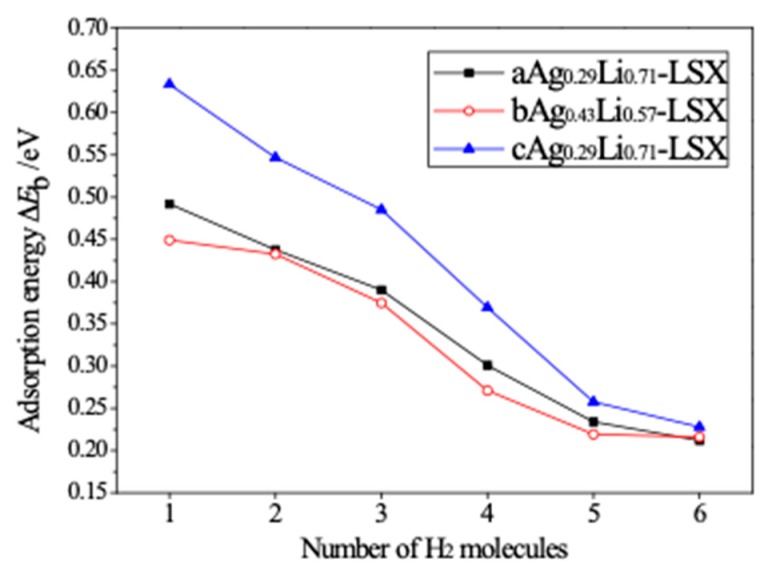
Adsorption binding energy of multiple H_2_ molecules around Ag cations for aAg_0.29_Li_0.71_-LSX, bAg_0.43_Li_0.57_-LSX and cAg_0.29_Li_0.71_-LSX, varying with the number of H_2_ molecules binding around one cation.

In order to investigate multiple H_2_ absorption mechanism, several H_2_ molecules are cumulatively placed one by one near the Ag cation cluster in the 3D unit cell of Ag-exchange Li-LSX, and then the model of multi-molecular H_2_ adsorption is established by geometric optimization of minimizing energy functional from first-principles calculations. The adsorption binding energy of added *n*^th^ H_2_ molecule is calculated using the following relation:

(3) $$E_{ad}=E[{\mathrm{Ag}}_{x}{\mathrm{Li}}_{1-x}\mathrm{LSX}+(n-{1)H}_{2}]+E(H_{2})-E({\mathrm{Ag}}_{x}{\mathrm{Li}}_{1-x}\mathrm{LSX}+nH_{2})$$

where *n* is number of absorbed H_2_ molecules and the constituent of adsorption system is denoted individually in bracket. The adsorption binding energy of multiple H_2_ molecules decreases evidently with the increase of H_2_ loading in Ag-exchange Li-LSX, demonstrating the repulsive interaction between H_2_ molecules and heterogeneous adsorption configuration on Ag-cluster cations, as the results shown in Figure 9. Furthermore, when the adsorption binding energy of H_2_ molecule declines to the lower limit of physical adsorption, it is implying that the H_2_ loading is saturated and approaching to uptake capacity.

**Figure 10:**
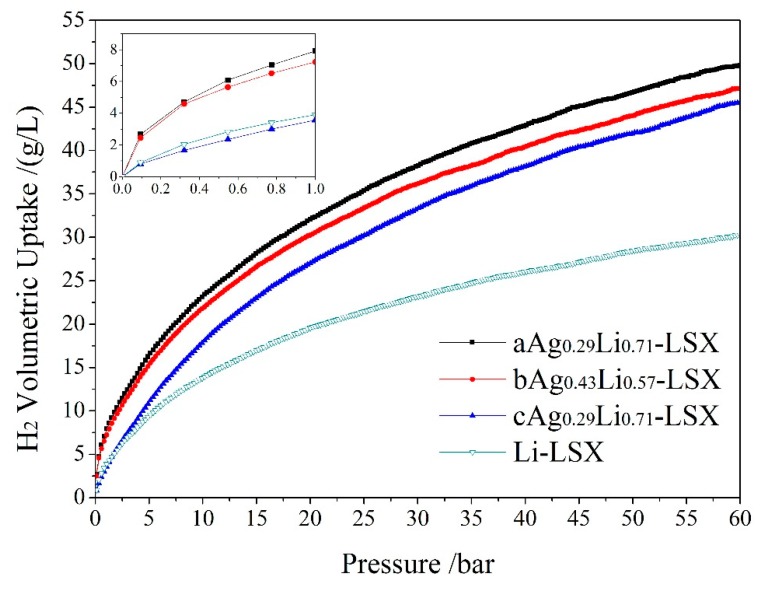
Metropolis Monte Carlo (MMC) simulated adsorption isotherms of H_2_ volumetric uptake by Li-LSX and Ag-exchange Li-LSX in comparison at 77 K. The inset specifies the H_2_ uptakes in pressure range of 0.0–1.0 bar.

Based on the results of the first-principles calculations, the adsorption of H_2_ by Ag-exchange Li-LSX is further simulated by using the MMC method as implemented with Sorption code to calculate the isothermal curves of H_2_ adsorption (absorption isotherm), as the results shown in Figure 10 for the H_2_ uptakes of Li-LSX, aAg_0.29_Li_0.71_-LSX, bAg_0.43_Li_0.57_-LSX and cAg_0.29_Li_0.71_-LSX as a function of pressure at 77 K temperatures. It is proved that the modification of Li-LSX by doping Ag can effectively improve the H_2_ storage capacity, especially at high pressure. When the pressure increases to 5 bar, the H_2_ uptakes of aAg_0.29_Li_0.71_-LSX and bAg_0.43_Li_0.57_-LSX increases to ~16.4 g/L, which is significantly higher than 9.2 g/L of Li-LSX. Due to the extremely high free volume of Ag-exchange Li-LSX and Li-LSX (81.3%), the H_2_ uptake rises continuously with the increasing pressure, and is still not saturated when the pressure increases to 60 bar, approaching to ~47.5 g/L and 30.3 g/L respectively, as shown in Figure 10. The simulated results of H_2_ absorption (absorption mass per unit volume) of Ag-doped and undoped Li-LSX are listed in Table 3, in comparison with the experimental results of several typical nano-porous materials reported in literature. H_2_ uptake of Ag-exchange Li-LSX at 77K temperature and 1bar pressure reaches 8.01 g/L, twice as much as 3.87 g/L of Li-LSX, which is comparable to the Metal-organic framework (MOF) with high H_2_ storage capability. On the other hand, the density of Ag-exchange Li-LSX is obviously higher than that of Li-LSX owing to the much higher mass of Ag atom than Li atom, hence H_2_ absorption calculated by mass ratio is close to or even lower than that of Li-LSX. Moreover, aAg_0.29_Li_0.71_-LSX or bAg_0.43_Li_0.57_-LSX can storage appreciably more H_2_ than cAg_0.29_Li_0.71_-LSX at lower pressure (<10 bar), as shown in Figure 10. Due to the larger space around the Ag cation inside supercages of aAg_0.29_L_i0.71_-LSX or bAg_0.43_Li_0.57_-LSX than the Ag cations in sodalite cages as for cAg_0.29_Li_0.71_-LSX, the Ag cation substituting Li^+^ on a sites can effectively bind more H_2_ molecules, resulting higher H_2_ uptake. It is reasonably verified from first-principle calculations and MMC simulations that the H_2_ volumetric uptake will be remarkably increased by Ag ion exchange, prospectively suggesting that Ag-exchange Li-LSX will be a promising candidate of nanoporous material in application for future H_2_ storage.

## 4. Conclusions

The magnetic source, electrochromic and H_2_ adsorption mechanism of Ag doped FAU zeolites (Ag-exchange Li-LSX) are theoretically studied by calculating the crystal structure, spin polarized electron structure, optical absorption/reflectance spectra, and H_2_ storage characteristics through first-principles schemes and MMC simulations. It is proved that Ag-exchange Li-LSX are direct band-gap semiconductors and represent considerable magnetism and good electrochromic properties. The CBM characteristics are mainly determined by the *p*-orbitals of Ag atom. With the increase of the Ag content, the electronic *p*-states of exchange Ag cations move to higher energy, resulting in a wider optoelectronic band-gap. The calculated results of spin polarization indicate that there is a major ferromagnetic spin-coupling between the Ag cations which are also ferrimagnetic-coupling with zeolite framework, resulting in a clear net magnetic moment. The spin polarization of Ag-4*d* orbital electrons essentially contributes to the magnetism of Ag-exchange Li-LSX. The calculated optical properties exhibit discriminative characteristics of optical absorption and reflection in visible region under different charged states, implying remarkable electrochromic performance that can be used as an electrical-color material for charge detection in monitoring power equipment.

The first-principles calculated adsorption structure and binding energy of H_2_ molecules in Ag-exchange Li-LSX demonstrate that H_2_ adsorption can significantly be enhanced through doping Ag in Li-LSX. However, the binding energy of H_2_ molecules on the Ag cations substituting Li cations in sodalite cages of Al-O-Si framework exceeds the lower limit of chemisorption energy, leading to the difficulty in reversible H_2_ desorption at room temperature. Nevertheless, the interaction of H_2_ molecule with the Ag cations in supercages is intensive physical adsorption while below chemical adsorption, so that the reversible process of adsorption/desorption can be fulfilled at ambient temperature. Therefore, the reasonable doping Ag by intentionally exchanging Li cations on the porous surface inside supercage of Li-LSX is an efficient routine to improve the H_2_ storage performance. MMC molecular simulations further proves that Ag doping can effectively increase the H_2_ uptake of Li-LSX due to the strong binding between H_2_ molecules and Ag cations, especially at low pressure. At 77 K temperature and 1 bar pressure, the H_2_ absorption of aAg_0.29_Li_0.71_-LSX and cAg_0.29_Li_0.71_-LSX reaches 8.01 and 7.25 g/L respectively, which are evidently higher than that of Li-LSX and comparable to the representative MOF with high H_2_ storage capability. The H_2_ storage capacities of Ag-exchange Li-LSX and Li-LSX increase continuously with increasing pressure up to >60 bar without saturated nature due to the extremely high free volume. TM doping as for Ag ion exchange provides an effective modification scheme to achieve high H_2_ storage performance for nanoporous materials of FAU zeolites.

## Figures and Tables

**Table 1:** Atomic structures from first-principles calculations: lattice constant a, coordinating distances of metallic cations with zeolite framework *d*_Li-O_ and *d*_Ag-O_, and atomic bonding length and angles in Al-Si-O cages, with the available experimental data of Li-LSX below for comparison [24].

Materials		Lattice Constant/Å	*d*_Li-O_/Å	*d*_Ag-O_/Å	Bonding Length/Å		Bonding Angle/(°)		
					Al–O	Si–O	O–Al–O	O–Si–O	Al–O–Si
aAg_0.29_Li_0.71_LSX		17.64	1.92, 1.90	2.44	1.75–1.79	1.62–1.66	105.4–113.3	105.0–113.0	128.5–138.5
bAg_0.43_Li_0.57_LSX		17.63	1.96, 1.90	2.42	1.74–1.79	1.62–1.66	106.1–116.6	105.0–113.0	125.5–145.1
cAg_0.29_Li_0.71_LSX		17.48	1.90, 1.92	2.552	1.62–1.78	1.62–1.65	104.3–114.5	105.4–112.7	121.6–146.5
acAg_0.57_Li_0.43_LSX		17.66	1.92	2.27, 2.64	1.75–1.79	1.62–1.66	105.0–113.4	105.1–113.1	127.9–141.6
bcAg_0.71_Li_0.29_LSX		17.56	1.92	2.32, 2.60	1.74–1.78	1.62–1.65	104.6–115.1	105.6–113.2	128.5–147.8
abAg_0.71_Li_0.29_LSX		18.05	1.98	2.29, 2.46	1.76–1.79	1.63–1.66	105.4–111.7	106.5–111.3	135.9–149.5
Li-LSX	Theor	17.52	1.93, 1.98, 1.91	–	1.71–1.78	1.61–1.66	104.7–113.4	105.3–112.3	122.7–142.6
	Exp [24]	17.45	1.98, 2.00, 1.89	–	1.66–1.76	1.58–1.72	105.0–112.8	106.7–110.8	125.6–143.8

**Table 2:** The calculated values of electronic band-gaps and net spins for Ag-exchange Li-LSX.

Materials	Optical Band-Gap/eV	Net Spin/(*ћ*/2)
aAg_0.29_Li_0.71_-LSX	1.70	3.92
bAg_0.43_Li_0.57_-LSX	1.93	−2.06
cAg_0.29_Li_0.71_-LSX	3.37	0
acAg_0.57_Li_0.43_-LSX	1.85	3.95
bcAg_0.71_Li_0.29_-LSX	2.16	−2.21
abAg_0.71_Li_0.29_-LSX	1.96	1.99

**Table 3:** H_2_ uptakes in newly reported porous materials for comparison.

Materials	Uptake/(g/L) at *T* = 77 K			
	*p* = 1 bar	*p* = 20 bar	*p* = 30 bar	*p* = 50 bar
Li-LSX	3.87	19.63	27.23	28.65
aAg_0.29_Li_0.71_-LSX	8.01	32.00	38.37	46.71
bAg_0.43_Li_0.57_-LSX	7.25	30.29	36.21	44.05
cAg_0.29_Li_0.71_-LSX	3.64	27.10	23.24	42.07
IRMOF-Hs [25]	5.41–9.79	23.90–32.81		29.72–37.36
CA-carbon [26]	18.67–22.71	38.77–45.43	40.32–49.09	
ZIF-8 [27]		28.45		30.41
Mg-MOF-74 [27]		35.95		33.98
